# The Molecular Pathogenesis of Tumor-Suppressive *miR-486-5p* and *miR-486-3p* Target Genes: *GINS4* Facilitates Aggressiveness in Lung Adenocarcinoma

**DOI:** 10.3390/cells12141885

**Published:** 2023-07-18

**Authors:** Yuya Tomioka, Takayuki Suetsugu, Naohiko Seki, Kengo Tanigawa, Yoko Hagihara, Masahiro Shinmura, Shunichi Asai, Naoko Kikkawa, Hiromasa Inoue, Keiko Mizuno

**Affiliations:** 1 Department of Pulmonary Medicine, Graduate School of Medical and Dental Sciences, Kagoshima University, Kagoshima 890-8544, Japan; k4829264@kadai.jp (Y.T.); taka3741@m2.kufm.kagoshima-u.ac.jp (T.S.); k8802984@kadai.jp (K.T.); k5382596@kadai.jp (Y.H.); k6271399@kadai.jp (M.S.); inoue@m2.kufm.kagoshima-u.ac.jp (H.I.); keim@m.kufm.kagoshima-u.ac.jp (K.M.); 2 Department of Functional Genomics, Chiba University Graduate School of Medicine, Chuo-ku, Chiba 260-8670, Japan; naoko-k@hospital.chiba-u.jp; 3 Head and Neck Surgery, Chiba Cancer Center, Nitona, Chiba 260-8717, Japan; sasai@chiba-cc.jp

**Keywords:** microRNA, passenger strand, *miR-486-5p*, *miR-486-3p*, *GINS4*, lung adenocarcinoma

## Abstract

**Simple Summary:**

Two microRNAs (miRNAs) (*miR-486-5p* and *miR-486-3p*) derived from pre-*miR-486* acted as tumor-suppressive miRNAs in lung adenocarcinoma (LUAD). We identified seven genes (*MKI67*, *GINS4*, *RRM2*, *HELLS*, *MELK*, *TIMELESS*, and *SAPCD2*) involved in the malignant phenotype of LUAD cells coordinately regulated by these miRNAs. It is possible to suppress the malignant transformation of LUAD by controlling these genes.

**Abstract:**

The involvement of passenger strands of miRNAs in the molecular pathogenesis of human cancers is a recent concept in miRNA research, and it will broaden our understanding of the molecular mechanisms of miRNA-mediated cancer. The analysis of our miRNA signature of LUAD revealed that both strands of pre-*miR-486* (*miR-486-5p* and *miR-486-3p*) were downregulated in LUAD tissues. Ectopic expression of both miRNAs induced cell cycle arrest in LUAD cells, suggesting both strands of miRNAs derived from pre-*miR-486* were tumor suppressive. Our in silico analysis showed a total of 99 genes may be under the control of both miRNAs in LUAD cells. Importantly, among these targets, the high expression of seven genes (*MKI67*, *GINS4*, *RRM2*, *HELLS*, *MELK*, *TIMELESS*, and *SAPCD2*) predicted a poorer prognosis of LUAD patients (*p* < 0.05). We focused on *GINS4*, a DNA replication complex GINS protein that plays an essential role in the initiation of DNA replication. Our functional assays showed that *GINS4* was directly controlled by both strands of pre-*miR-486*, and its aberrant expression facilitated the aggressive behavior of LUAD cells. *GINS4* is attractive as a therapeutic target for this disease. MiRNA analysis, including passenger strands, will further improve our understanding of the molecular pathogenesis of LUAD.

## 1. Introduction

Lung cancer is the leading cause of cancer-related deaths in men and women worldwide, with approximately 2.3 million people diagnosed with lung cancer and approximately 1.8 million deaths from lung cancer each year [1]. Lung cancer is divided histologically into two groups: small cell lung cancer (SCLC), which accounts for 15% of patients, and non-SCLC (NSCLC), which accounts for 85% [2]. NSCLC are subdivided into squamous cell carcinoma (LUSQ), large cell carcinoma, and lung adenocarcinoma (LUAD); the latter accounts for approximately 60% of NSCLC [2].

The curative treatment for lung cancer patients at an early stage of the disease (stage I or II) is surgical resection; however, even those who undergo radical surgery have a 5-year survival rate of only 65% [3]. However, fewer than 30% of lung cancer patients are diagnosed early in the course of disease and proceed to surgical treatment. Patients diagnosed with advanced-stage lung cancer have a very poor prognosis, with only 20% of patients surviving 5 years [2,4].

Therapeutic strategies for advanced stage LUAD are developing in a remarkable way. Molecular-targeted drugs that counteract driver gene alteration (e.g., *EGFR* mutation, *ALK* rearrangement, *ROS1* rearrangement, *BRAF* mutation, *MET* mutation, and *KRAS* mutation), and immune checkpoint inhibitors are showing therapeutic effects [5,6]. However, the prognosis for lung cancer patients remains extremely poor, with only 20% of those diagnosed at an advanced stage surviving for five years [2,4]. Therefore, the search for new diagnostic biomarkers and therapeutic target molecules is an important research theme for improving the prognosis of patients with LUAD.

Numerous non-coding RNAs (ncRNAs) are involved in a wide variety of biologic functions, e.g., basic metabolism and cell differentiation. At present, ncRNAs are thought to play important roles for maintaining cellular homeostasis [7,8,9]. A large number of studies have shown that various ncRNAs are aberrantly expressed in diverse tumors, indicating that such RNAs play important roles in tumorigenesis and development [10,11,12]. Among ncRNAs, miRNAs are small, single-stranded ncRNAs (19–22 nucleotides in length). They act as fine tuners of gene expression and modulate almost all biological processes [13,14]. Aberrant expressions of miRNAs are frequently detected in a wide range of cancers, including LUAD. Aberrant-expressed miRNAs are closely involved in the malignant transformation of human cancers, e.g., proliferation, metastasis, and resistance [15,16].

In the previous concept of miRNA biogenesis, only the guide strand of miRNAs derived from pre-miRNAs were actually functional miRNAs in cells. On the other hand, the passenger strand was thought to be broken down inside the cell and to have no function. In contrast to the miRNA theory, some passenger strands of miRNAs have been shown to regulate their target molecules [17,18]. These studies indicate that an miRNA analysis of gene regulation requires the inclusion of both the guide and passenger strands. For example, our recent studies on NSCLC cells demonstrated that both strands of *miR-99a*, *miR-144*, *miR-145*, and *miR-150*, had tumor-suppressive activity through their control of several oncogenic genes [19,20,21,22]. Based on these studies, we hypothesized that genes that are commonly regulated by both strands derived from pre-miRNA are highly involved in the oncogenesis of LUAD.

To identify dysregulated miRNAs in LUAD clinical tissues, we generated miRNA expression signatures by using small RNA sequencing technology. The analysis of the signatures revealed that both strands of pre-*miR-486* (*miR-486-5p*, the guide strand, and *miR-486-3p*, the passenger strand) were downregulated in LUAD tissues. Moreover, they acted as antitumor miRNAs in our functional assays. Importantly, seven genes (*MKI67*, *GINS4*, *RRM2*, *HELLS*, *MELK*, *TIMELESS*, and *SAPCD2*) commonly regulated by *miR-486-5p* and *miR-486-3p* were closely involved in the molecular pathogenesis of LUAD. Furthermore, the aberrant expression of *GINS4*, a DNA replication complex GINS protein, facilitated LUAD cell aggressiveness.

Our signature-based miRNA analysis accelerates the discovery of genes closely involved in LUAD tumorigenesis. These genes are potential therapeutic targets for this disease.

## 2. Materials and Methods

### 2.1. Clinical Course of Patients with LUAD Cells

We obtained primary lesions and normal lung tissues from lung adenocarcinoma patients. The background and clinical characteristics of the patients are described in Table S1.

### 2.2. Cell Lines and Cell Culture 

Two LUAD cell lines, A549 and H1299, were used in this study (American-Type Culture Collection (ATCC), Manassas, VA, USA). We have previously described the method of cell maintenance [21,23].

### 2.3. Construction of the miRNA Expression Signature in LUAD Based on RNA Sequencing

LUAD and normal lung specimens were sequenced using a the NextSeq 500 instrument (Illumina, Inc., San Diego, CA, USA) to evaluate miRNA expression. The raw sequencing data were registered in Gene Expression Omnibus (GEO; GEO accession number: GSE230229).

### 2.4. Identification of Oncogenic Targets Regulated by miR-486-5p and miR-486-3p in LUAD Cells

We used the expression profiles of genes from A549 cells transfected with *miR-486-5p* or *miR-486-3p* (GEO accession number: GSE230056) and TargetScanHuman ver.8.0 (https://www.targetscan.org/vert_80/, accessed on 12 January 2023) to search for miRNAs regulated by *miR-486-5p* and *miR-486-3p*.

### 2.5. Expression Levels of Genes and Prognosis by In Silico Analysis

The clinical significance of genes in LUAD was evaluated with The Cancer Genome Atlas (TCGA) datasets (https://www.cancer.gov/tcga, accessed on 17 January 2023). The data describing gene expression levels were obtained from FIREBROWSE (http://firebrowse.org/, accessed on 17 January 2023) and Genomic Data Commons (GDC) Data Portal (https://portal.gdc.cancer.gov/, accessed on 17 January 2023). The overall survival data were obtained from cBioPortal (https://www.cbioportal.org/, accessed on 17 January 2023) and OncoLnc (http://www.oncolnc.org/) (data downloaded on 17 January 2023).

### 2.6. Transfection with siRNA and miRNA

siRNA and miRNA were transfected into cell lines using Opti-MEM (catalog no.: 31985070, Gibco, Carlsbad, CA, USA) and LipofectamineTM RNAiMax Transfection Reagent (catalog no.: 13778150, Invitrogen, Carlsbad, CA, USA). Transfection protocols for siRNA and miRNA were described in our previous studies [21,23,24]. siRNA and miRNA used in this study are listed in Table S2.

### 2.7. RNA Extraction and Reverse Transcription Quantitative Polymerase Chain Reaction (RT-qPCR)

Total RNA obtained from LUAD cell lines was isolated using Isogen II (catalog no.: 311-07361, NIPPON GENE Co., Ltd., Tokyo, Japan). cDNA was synthesized using PrimeScriptTM RT Master Mix (catalog no.: RR036A, Takara Bio Inc., Shiga, Japan). Gene expression was analyzed by real-time PCR using a SYBR green assay (ThermoFisher Scientific, Rockford, IL, USA) on a StepOnePlus Real-Time PCR System (Applied Biosystems, Foster City, CA, USA). The internal control used in the gene expression assays was glyceraldehyde 3-phosphate dehydrogenase (GAPDH). The reagents used in this study are listed in Table S2.

### 2.8. Western Blotting 

LUAD cells were lysed with the RIPA Lysis Buffer System (catalog no.: sc-24948, Santa Cruz Biotechnology Inc., Dallas, TX, USA). Protein concentrations were measured using a PierceTM BCA Protein Assay Kit (catalog no.: 23227, Thermo Fisher Scientific, Rockford, IL, USA). We used SuperSepTM Ace (7.5%, 13 well) (catalog no.: 198-14941, FUJIFILM Wako Pure Chemical Corporation, Osaka, Japan) as the SDS-PAGE gel for electrophoresis and Precision Plus ProteinTM Dual Color Standards (catalog no.: #1610374, Bio-Rad Laboratories, Inc., Hercules, CA, USA). The proteins were transferred to polyvinylidene fluoride membranes (catalog no.: PPVH00010, Merck KGaA, Darmstadt, Germany). The membranes were blocked with 5% skimmed milk (catalog no.: 190-12865, FUJIFILM Wako Pure Chemical Corporation, Osaka, Japan) in TBST. The signal was detected using Amersham ECL Prime Western Blotting Detection Reagent (Cytiva, Marlborough, MA, USA). The reagents used in this study are listed in Table S2.

### 2.9. Cell Proliferation and Cell Cycle Assays

Cell proliferation was evaluated with XTT assays using Cell Proliferation Kits (catalog no.: 20-300-1000, Biological Industries, Beit-Haemek, Israel). The cell cycle was evaluated using a BD CycletestTM Plus DNA Reagent Kit (catalog no.: 340242, BD Biosciences, Franklin Lakes, NJ, USA) and flow cytometry (BD FACSCelestaTM Flow Cytometer, BD Biosciences). The procedures for assessing cell proliferation and cell cycle behaviors were described previously [21,23,24].

### 2.10. Plasmid Construction and Dual-Luciferase Reporter Assays

The following two sequences were cloned into the psiCHECK-2 vector (C8021; Promega, Madison, WI, USA): the wild-type sequence of the 3′-untranslated regions (UTRs) of *GINS4* and the deletion-type sequence, which lacked the *miR-486-5p* and *miR-486-3p* target sites of *GINS4*. The procedures for the transfection and dual-luciferase reporter assays were provided previously [21,23,24].

### 2.11. Immunohistochemical Staining

*GINS4* expression was evaluated by immunohistochemical staining using tissue microarray slides (catalog no.: LC811a, US Biomax, Inc. Derwood, MD, USA). The VECTASTAIN Universal Elite ABC Kit (catalog no.: PK-6200, Vector Laboratories, Burlingame, CA, USA) was used for blocking, the primary antibody reaction, the secondary antibody reaction, and the binding of avidin to the biotin complex. Primary antibodies were diluted with Dako Real antibody diluent (catalog no.: K5007, Agilent, Santa Clara, CA, USA). Dako REALTM EnVisionTM Detection System Peroxidase/DAB+, Rabbit/Mouse (Agilent) was used to develop the chromogenic reaction. The primary antibody used in this study is described in Table S2. Clinical tissue information is presented in Table S3.

### 2.12. Putative miRNA Binding to GINS4 and miRNA Expression 

We obtained the data for putative miRNA binding to *GINS4* from TargetScanHuman database (release 8.0) and the data for miRNA expression levels from FIREBROWSE (http://firebrowse.org/, accessed on 31 October 2022) and Genomic Data Commons (GDC) Data Portal (https://portal.gdc.cancer.gov/) (accessed on 31 October 2022).

### 2.13. Statistical Analysis

Statistical analyses were performed using GraphPad Prism 8 (GraphPad Software, La Jolla, CA, USA) and R ver. 4.2.1 (R Core Team, Vienna, Austria; https://www.R-project.org/, accessed on 3 September 2022). The differences between 2 groups were analyzed by Student’s *t*- or Mann–Whitney U tests. Multiple group comparison was achieved using a one-way analysis of variance (ANOVA) and Tukey’s tests for post hoc analysis. Survival rates were analyzed by Kaplan–Meier survival curves and the log-rank test.

## 3. Results

### 3.1. Selection of Downregulated miRNAs in LUAD Clinical Specimens by Small RNA Sequencing 

To create the miRNA expression signatures of LUAD, we prepared nine cDNA libraries obtained from clinical specimens (five LUAD tissues and four normal lung tissues) and performed RNA sequencing. The clinical information for the LUAD tissues is summarized in Table S1. The processing of the data based on the RNA sequencing analysis and details of analyzed small RNA taxonomies are presented in Table S4. We successfully mapped a sufficient number of miRNA reads to the human genome (Table S4).

**Figure 1:**
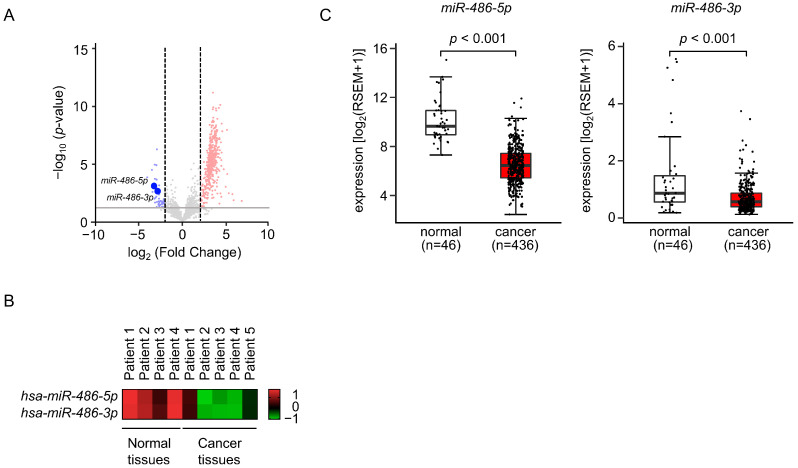
Expression levels of *miR-486-5p* and *miR-486-3p* in LUAD clinical specimens. (**A**) Volcano plot of the miRNA expression signature determined through RNA sequencing. The log_2_ fold-change (FC) is plotted on the *x*-axis and the log_10_ (*p*-value) is plotted on the *y*-axis. The blue points represent the downregulated miRNAs with an absolute log_2_ FC < 2.0 and *p* < 0.05. The red points represent the upregulated miRNAs with an absolute log_2_ FC > 2.0 and *p* < 0.05. Our miRNA expression data by RNA sequencing are deposited in the GEO database (accession number: GSE230229). (**B**) Heat map of the expression levels of *miR-486-5p* and *miR-486-3p* for normal lung and LUAD tissues based on the LUAD miRNA signature obtained by RNA sequencing. (**C**) The expression levels of *miR-486-5p* and *miR-486-3p* evaluated in an LUAD dataset from TCGA.

We analyzed RNA sequence data (a total of 41 miRNAs) in the LUAD tissues for comparison with normal lung tissues (Table 1, Figure 1A). We found they were significantly downregulated (log_2_ fold-change < −2.0 and *p*-value < 0.05). Interestingly, our signature revealed that both the guide and passenger strands of four miRNAs (*miR-34c*, *miR-486*, *miR-34b*, and *miR-144*) were downregulated in the LUAD tissues (Table 1). The involvement of passenger strands of miRNAs derived from pre-miRNAs in the molecular pathogenesis of human cancers is a recent concept in miRNA biology. 

### 3.2. Expression Levels of miR-486-5p and miR-486-3p in LUAD Specimens and Cell Lines

To confirm our miRNA signature, we evaluated the expression levels of *miR-486-5p* and *miR-486-3p* in LUAD tissues and normal lung tissues.

Both *miR-486-5p* and *miR-486-3p* were significantly downregulated in LUAD tissues (Figure 1B). The TCGA dataset analysis confirmed that the expression levels of *miR-486-5p* (*p* < 0.001) and *miR-486-3p* (*p* < 0.001) were significantly lower in the LUAD tissues (*n* = 436) compared to normal tissues (*n* = 46) (Figure 1C).

### 3.3. Antitumor Functions of miR-486-5p and miR-486-3p in LUAD Cells

In order to prove that both strands of pre-*miR-486* had antitumor functions in LUAD cells, we performed an ectopic expression of these miRNAs in LUAD cells (A549 and H1299), and then investigated the behavior of the cancer cells.

**Figure 2:**
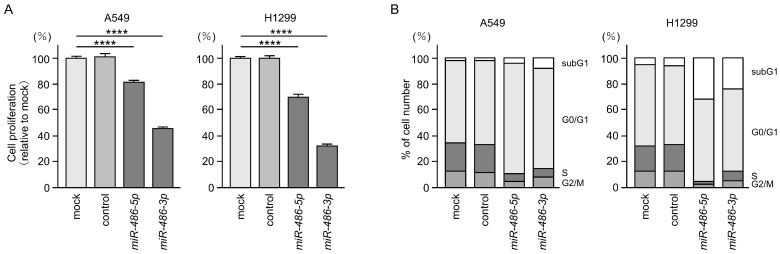
Antitumor roles of *miR-486-5p* and *miR-486-3p* in LUAD cells. (**A**) Cell proliferation was assessed using XTT assays 72 h after transfection with *miR-486-5p* or *miR-486-3p* in LUAD cells. (**B**) Cell cycle changes were analyzed by flow cytometry. Assays were performed 72 h after transfections with *miR-486-5p* or *miR-486-3p* in LUAD cells. ****, *p* < 0.0001.

Cancer cell proliferation was attenuated by the ectopic expression of *miR-486-5p* or *miR-486-3p* in LUAD cells (Figure 2A). The analysis showed typical cell cycle arrest (G0/G1 phase) after both miRNAs were transfected into LUAD cells (Figure 2B).

Based on these results, we conclude that the two types of miRNAs derived from pre-*miR-486* are tumor suppressive miRNAs in LUAD cells.

### 3.4. Identification of Genes Controlled by miR-486-5p and miR-486-3p in LUAD Cells

The fact that both miRNA strands derived from pre-*miR-486* acted as antitumor miRNAs was quite interesting. The subsequent challenge is to identify the oncogenic targets controlled by these miRNAs in LUAD cells.

**Figure 3:**
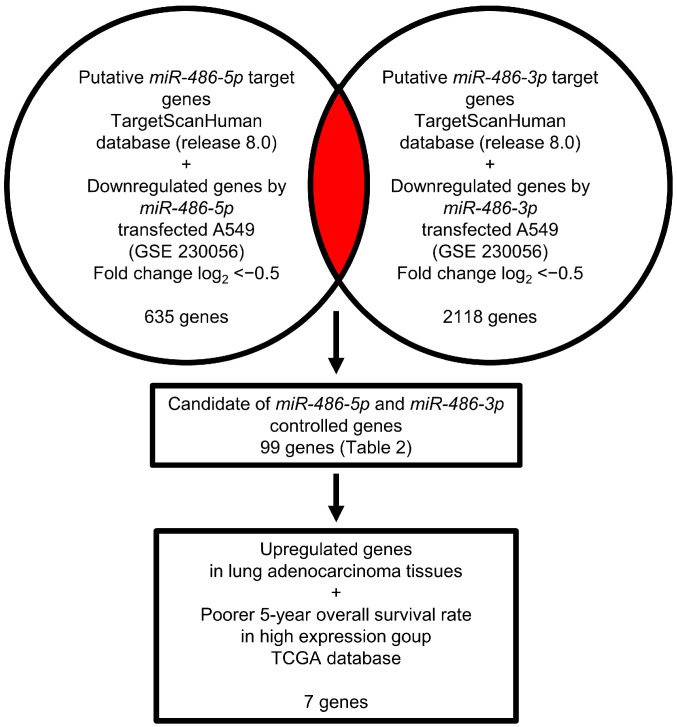
Strategy for identifying oncogenic targets subject to *miR-486-5p* and *miR-486-3p* common regulations in LUAD cells. To identify *miR-486-5p* or *miR-486-3p* targets, we used the TargetScanHuman (release 8.0) database and gene expression profiles generated after *miR-486-5p* or *miR-486-3p* were transfected into A549 cells. Our original gene expression array data were deposited in the GEO database (accession number: GSE230056). In total, 99 genes were identified as possibly being controlled by both *miR-486-5p* and *miR-486-3p* in A549 cells.

Our strategy for the search for miRNAs targets is shown in Figure 3. In this study, we obtained genome-wide gene expression data using *miR-486-5p*- or *miR-486-3p*-transfected A549 cells. Our gene expression data were deposited in the GEO database (accession number: GSE230056).

Using a combination of the TargetScan database and miRNA-transfected LUAD cells expression data, we searched for putative targets controlled by *miR-486-5p* (635 genes) and *miR-486-3p* (2118 genes). Notably, 99 genes were shown to be potential targets of both *miR-486-5p* and *miR-486-3p* in LUAD cells (Table 2).

### 3.5. Expression and Clinical Significance of Both Strands of Pre-miR-486 Target Genes in LUAD

A further analysis of these 99 genes was performed to search for those that promoted cancer in LUAD cells.

**Figure 4:**
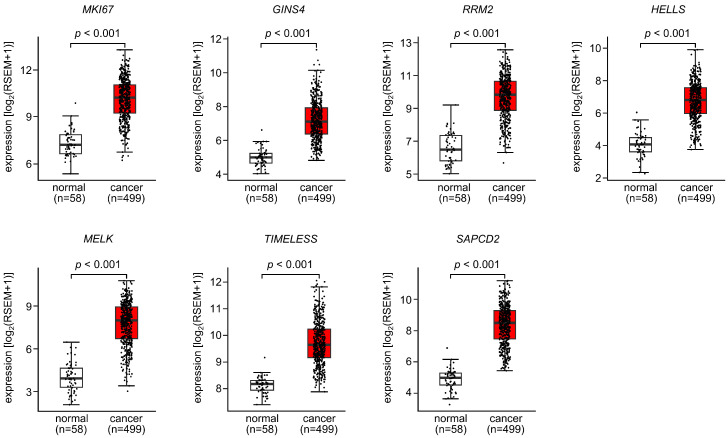
Expression levels of putative target genes controlled by both *miR-486-5p* and *miR-486-3p* in LUAD clinical specimens. Among the 99 putative targets (Table 2), 7 target genes (*MKI67*, *GINS4*, *RRM2*, *HELLS*, *MELK*, *TIMELESS*, and *SAPCD2*) were upregulated in LUAD clinical specimens using TCGA-LUAD datasets.

We validated the expression levels of these 99 target genes using a large amount of clinical data (TCGA-LUAD). The expression of seven genes (*MKI67*, *GINS4*, *RRM2*, *HELLS*, *MELK*, *TIMELESS*, and *SAPCD2*) was upregulated in the LUAD tissues (*n* = 499) compared with normal lung tissues (*n* = 58) (Figure 4).

**Figure 5:**
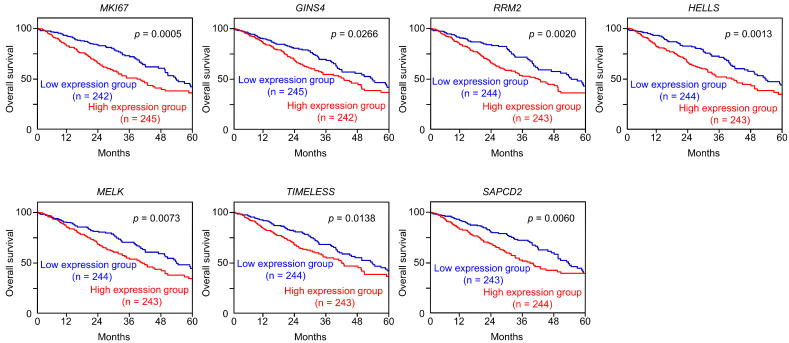
Clinical significance of 7 target genes (*MKI67*, *GINS4*, *RRM2*, *HELLS*, *MELK*, *TIMELESS*, and *SAPCD2*) in LUAD clinical specimens. Kaplan–Meier curves of the five-year overall survival rates according to the expression levels of each gene. The low expression levels of all seven genes were significantly predictive of a poorer prognosis in patients with LUAD. The patients were divided into two groups—high- and low-expression groups—according to the median gene expression level. The red and blue lines represent high- and low-expression groups, respectively.

A clinicopathological analysis of the seven genes was performed using TCGA-LUAD datasets. Kaplan–Meier curve (5-year survival rates) analysis was performed according to the expression levels of the seven genes. The high expression of all genes significantly affected the poorer survival rates of the patients (Figure 5).

### 3.6. Direct Regulation of GINS4 by miR-486-5p and miR-486-3p in LUAD Cells

First, we investigated whether the expression of the seven selected genes was controlled by *miR-486-5p* and *miR-486-3p* in LUAD cells. The mRNA expression levels of all seven genes were remarkably suppressed in *miR-486-3p*-transfected A549 cells (Figure S1). In *miR-486-5p*-transfected cells, the expression levels of five genes (*MKI67*, *GINS4*, *RRM2*, *HELLS* and *MELK*) were significantly suppressed (Figure S1).

In our previous analysis, we focused on the genes involved in DNA replication [25,26]. In this study, we focused on *GINS4* and investigated the oncogenic roles of its aberrant expression in LUAD cells.

**Figure 6:**
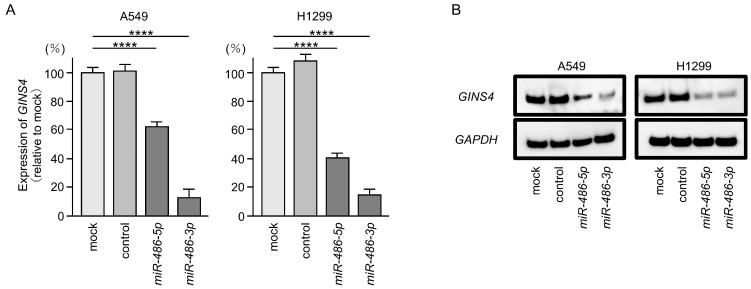
Ectopic expression levels of *miR-486-5p* and *miR-486-3p* reduced the expression level of *GINS4* in LUAD cells. (**A**) Expression levels of *GINS4* mRNA were significantly reduced in *miR-486-5p*- or *miR-486-3p*-transfected cells (A549 and H1299). Total RNAs were extracted 72 h after miRNA transfection and measured by real-time PCR methods. *GAPDH* was used as an internal control. The experiment was performed 3 times, with one-way ANOVA and Tukey’s tests for the post hoc analysis. ****, *p* < 0.001 (**B**) Expression levels of GINS4 proteins were significantly reduced in *miR-486-5p-* or *miR-486-3p*-transfected cells (A549 and H1299). Proteins were extracted 72 h after miRNAs transfection and measured by Western blotting methods. GAPDH was used as an internal control.

We confirmed that *GINS4* expression in LUAD cells was suppressed at the mRNA and protein levels by the ectopic expression of *miR-486-5p* or *miR-496-3p* (Figure 6A,B). Full-size images of Western blots are shown in Figure S2.

**Figure 7:**
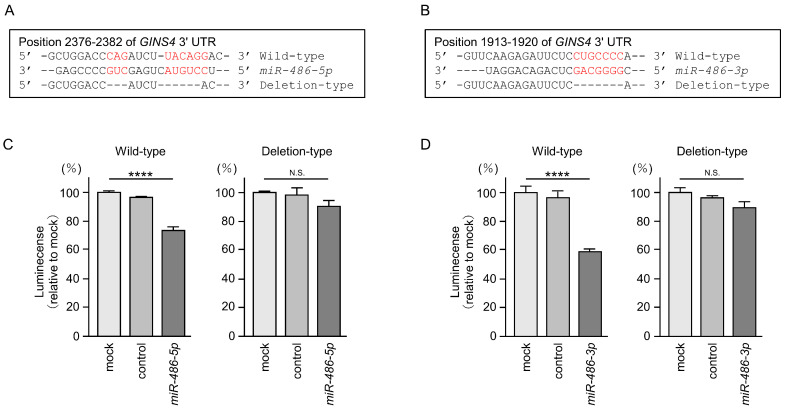
*miR-486-5p* and *miR-486-3p* were directly bound to the 3′-UTR of *GINS4* in LUAD cells. (**A**,**B**) Putative *miR-486-5p* and *miR-486-3p* binding sites on the 3′-UTR of the *GINS4* gene based on TargetScan database (release 8.0). (**C**,**D**) Dual-luciferase reporter assays showed reduced luminescence activity after co-transfection of the wild-type vector (containing the *miR-486-5p* binding site) with *miR-486-5p* in A549 cells. In contrast, no luminescence activity was seen after the co-transfection of the deletion-type vector (lacking the *miR-486-5p* binding site) with *miR-486-5p* in A549 cells. Similar analytic results were obtained for wild- or deletion-type vectors (with or without the *miR-486-3p* binding site) and *miR-486-3p* in A549 cells. ****, *p* < 0.001; N.S., not significant.

To demonstrate that both miRNAs directly bound to the 3′-UTR of the *GINS4* gene in a sequence-dependent manner, we performed dual-luciferase reporter assays. The putative *miR-486-5p* binding site on the 3′-UTR of the *GINS4* gene is shown in Figure 7A. Luciferase activity was significantly reduced when co-transfected with *miR-486-5p* and a vector containing binding sites for the 3′-UTR of *GINS4* (Figure 7C). However, luciferase activity did not change when co-transfected with *miR-486-5p* and a vector lacking the *miR-486-5p* binding site (Figure 7C). Thus, *miR-486-5p* appeared to directly bind to the 3′-UTR of *GINS4* in a sequence-dependent manner.

We also investigated the sequence-dependent direct binding of *miR-486-3p* and the 3′-UTR of the *GINS4* gene. The putative *miR-486-3p* binding site on the 3′-UTR of the *GINS4* gene is shown in Figure 7B. Luciferase activity was significantly reduced when co-transfected with *miR-486-3p* and a vector containing binding sites for the 3′-UTR of *GINS4* (Figure 7D). However, there was no change after the co-transfection of miR-486-3p and a vector lacking the *miR-486-3p* binding site (Figure 7D). These results indicate that *miR-486-3p* directly binds to the 3′-UTR of *GINS4* in a sequence-dependent manner.

### 3.7. Functional Significance of GINS4 in LUAD Cells

To investigate the oncogenic function of *GINS4* in LUAD cells, we made use of knockdown assays with siRNAs that were transfected into LUAD cells (A549 and H1299). We evaluated the knockdown efficiency of several siRNAs (si*GINS4*-1, si*GINS4*-2, and si*GINS4*-3) for *GINS4*. Transient transfection with three types of siRNAs significantly reduced *GINS4* mRNA and protein expression in LUAD cells (Figures S3 and S4). 

**Figure 8:**
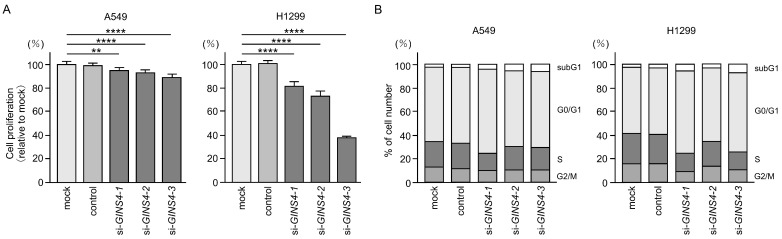
Effects of *GINS4* knockdown in LUAD cells. Three types of siRNAs (si*GINS4*-1, si*GINS4*-2, and si*GINS4*-3) were used for functional assays for the knockdown of *GINS4* expression. (**A**) Cell proliferation was assessed using an XTT assay 72 h after transfection with siRNAs (si*GINS4*-1 si*GINS4*-2, and si*GINS4*-3) in LUAD cells (A549 and H1299). ****, *p* < 0.0001; **, *p* < 0.05. (**B**) Cell cycle changes were analyzed by flow cytometry. Assays were performed 72 h after transfection with three types of siRNAs (si*GINS4*-1, si*GINS4*-2, and si*GINS4*-3) in LUAD cells (A549 and H1299).

LUAD cell proliferation assays showed that cell growth was inhibited by suppressing the expression of *GINS4* (Figure 8A). Moreover, cell cycle assays demonstrated that cell cycle arrest in the G0/G1 phase after the expression of *GINS4* was suppressed in LUAD cells (Figure 8B). These results suggest that *GINS4* is a cancer-promoting gene that modulates cell cycle progression.

### 3.8. Immunostaining of GINS4 in LUAD Clinical Tissues

**Figure 9:**
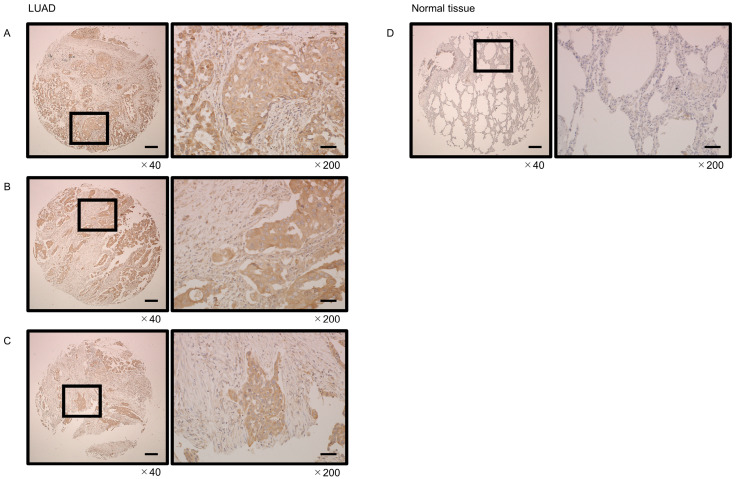
Expression of *GINS4* protein in LUAD clinical tissues assessed by immunostaining. (**A**–**C**) High expression of *GINS4* was detected in the cytoplasm of cancer lesions. (**D**) Weak expression of *GINS4* was detected in normal lung tissues. Scale bar: 200 µm (low magnification); 50 µm (high magnification).

We examined the expression of *GINS4* in tissue microarray studies. Compared with normal tissues, the *GINS4* protein was overexpressed in LUAD tissues. In particular, cancer cells showed heavy cytoplasmic staining (Figure 9).

### 3.9. GINS4-Mediated Pathways Determined by Gene Set Enrichment Analysis (GSEA)

To investigate *GINS4*-modulated molecular pathways in LUAD cells, we used the Gene Set Enrichment Analysis (GSEA) based on TCGA–LUAD RNA sequencing data.

**Figure 10:**
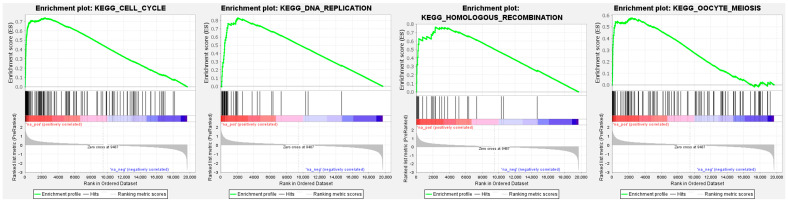
*GINS4*-mediated pathways identified by Gene Set Enrichment Analysis (GSEA). The top-4 enriched gene sets (enrichment plots) in patients in the high-*GINS4* group compared with the low-expression group.

“Cell cycle”, “DNA replication”, “homologous recombination”, and “oocyte meiosis” pathways were enriched in patients with high expression of *GINS4* compared to low-expression patients (Figure 10, Table 3).

## 4. Discussion

During classic miRNA maturation, pre-miRNA is transported to the cytoplasm where it is cleaved by Dicer to become a miRNA duplex. One strand derived from the miRNA duplex is incorporated into the RNA-Induced Silencing Complex (RISC), where it regulates specific target genes. That strand is defined as the guide strand. The non-loaded strand (the passenger strand) is degraded in the cytoplasm, as it has no known function [27]. However, in recent studies, both strands of the miRNA duplex were shown to be functional [27,28]. In this study, we confirmed that both strands of pre-*miR-486* had tumor suppressive functions by regulating their respective target genes.

*miR-486* is transcribed from the intron of the host gene *ANK1* (*Ankyrin 1*) on human chromosome 8p11.21 [29]. There have been many reports describing *miR-486-5p* (the guide strand) in various cancer types [30,31,32,33]. Previous studies showed that the expression of *miR-486-5p* was reduced in cancer tissues, and that it functions as a tumor suppressor in breast cancer, colorectal cancer, gastric cancer, hepatocellular carcinoma, and renal cell carcinoma [34]. In contrast to the preceding examples, the overexpression of *miR-486-5p* was observed in prostate cancer, and its expression is associated with the malignant transformation of these cancers [35].

In previous reports of lung cancer, the function of *miR-486-5p* was that of tumor suppression, which is consistent with our results [31]. For example, the expression of *miR-486-5p* blocked mTOR pathways through its targeting of ribosomal proteins S6 kinase A1 and B1 [36]. Moreover, the overexpression of *miR-486-5p* attenuated tumor growth and inhibited metastasis according to in vivo assays [36,37]. A recent study showed that the anesthetic propofol induced the expression of *miR-486-5p*, resulting in the inhibition of the Ras-associated protein1-NF-κB pathway [38]. These events contributed to cisplatin-sensitivity in NSCLC cells [38].

Several papers have reported that *miR-486-3p*, which is the passenger strand of pre-*miR-486*, has anti-tumor functions in several cancers [34]. In recent years, it has become clear that overexpression of various types of circular RNAs adsorbs miRNAs and suppresses their functions in normal and pathological cells [39,40]. Various circular RNAs are overexpressed in lung cancer cells, e.g., circ_EPB41, circ_CSPP1, and circ_0011298, and the adsorption of *miR-486-3p* by these circular RNAs promotes a malignant transformation [41,42,43]. In addition, these studies revealed that *eIF5A*, *BRD9*, *XRCC1*, *CYP1A1*, and *CRABP2* were *miR-486-3p*-regulated cancer-promoting genes in NSCLC cells [41,42,43,44]. The results of our functional analysis of *miR-486-3p* support those reports.

Since both strands of pre-*miR-486* are tumor suppressive, our subsequent interest is to elucidate the molecular networks regulated by these miRNAs in LUAD cells. Numerous studies have characterized the target molecules of miRNAs, including *miR-486-5p* and *miR-486-3p*; however, none have searched for common targets of these miRNAs in LUAD cells. Our study revealed that 99 genes were putative targets of *miR-486-5p* and *miR-486-3p* regulations in LUAD cells. In fact, all 99 genes were regulated by both *miR-486-5p* and *miR-486-3p* in LUAD cells. It should be noted that high expression of seven genes (*MKI67*, *GINS4*, *RRM2*, *HELLS*, *MELK*, *TIMELESS*, and *SAPCD2*) had a negative impact on the prognosis of patients with LUAD. These genes are important for elucidating the molecular mechanisms of lung cancer malignancy.

For these genes, we referred to reports of genes under microRNA regulation in lung cancer cells. The RRM2 protein is one of the two subunits of the ribonucleotide reductase complex. This reductase is a key enzyme in DNA synthesis as it catalyzes the formation of deoxyribonucleotides from ribonucleotides [45]. Previous studies showed that the overexpression of RRM2 was detected in a wide range of cancers, including lung cancer [45,46]. A recent study showed that the expression of *miR-203-3p* was reduced in LUAD tissues, and its overexpression inhibited LUAD aggressive phenotypes through targeting *RRM2* in LUAD cells [47]. Our previous study showed that *miR-150-3p* (the passenger strand) was significantly suppressed in LUSQ tissues, and performed a tumor-suppressive role in LUSQ cells via controlled several cell cycle-related genes, including *HELLS* [48]. HELLS belongs to the SNF2 family of chromatin-remodeling ATPases and is recruited to specific DNA sites to control the transcription of targeted genes [49,50]. Our data demonstrate that HELLS is directly regulated by *miR-150-3p* in LUSQ cells [48]. Previous reports revealed that SAPCD2 is highly expressed in various cancers and is highly involved in the malignant transformation of cancer cells [51]. Numerous studies have shown that SAPCD2 interacts with multiple proteins within the cell cycle interaction network and functions as a mitotic phase-promoting factor [51]. Notably, a recent study showed that *miR-486-5p* suppressed cell malignant progression in LUAD cells by targeting *SAPCD2* [52]. This fact is consistent with our data and indicates that *miR-486-5p*-mediated molecular networks have pivotal effects on LUAD cell malignancy.

Among these targets, we focused on *GINS4*, and we showed that its expression facilitated the malignant transformation of LUAD cells. *GINS4* is a member of the GINS complex, which consists of four different subunits, e.g., *GINS1* to *GINS4* [53]. Precisely maintained genomic DNA replication is critical for all forms of cellular life and requires a complex interplay of various protein factors. DNA helicases play a key role in unwinding double-stranded DNA during replication, recombination, and repair processes. The GINS complex forms the CMG (Cdc45-MCMs-GINS) complex with MCM (mini-chromosome maintenance) and CDC45. This complex functions as a replicative helicase that unwinds double-stranded DNA during chromosome replication [53,54].

The overexpression of *GINS4* occurs in breast cancer, colorectal carcinoma, bladder cancer, pancreatic ductal adenocarcinoma, glioma, and gastric cancer [55]. In NSCLC cells, lymphoid-specific helicase binds to the 3′-UTR region of *GINS4* and stabilizes *GINS4* expression [56]. The overexpression of *GINS4* facilitates lung cancer malignant transformation. The aberrant expression of other members of *GINS* (*GINS1*, *GINS2*, and *GINS3*) occurs in different types of human cancers [57,58]. The TCGA database analysis revealed that all members of *GINS* were upregulated in LUAD tissues and their high expression predicted the prognosis of the patients. Several studies demonstrated that the expression of a *GINS* member enhanced cancer cell aggressiveness, e.g., proliferation, drug resistance, and epithelial–mesenchymal transition [55,59,60]. Therefore, *GINS* members are closely involved with LUAD pathogenesis and may be potential therapeutic targets.

## 5. Conclusions

The analysis of the miRNA expression signature revealed that both strands of pre-*miR-486* (*miR-486-5p* and *miR-486-3p*) were downregulated in LUAD tissues. From this study and previous reports, we confirmed that these miRNAs had tumor-suppressive functions in LUAD cells. A total of 99 genes were identified as cooperatively controlled by *miR-486-5p* and *miR-486-3p* in LUAD cells. Among these targets, seven genes (*MKI67*, *GINS4*, *RRM2*, *HELLS*, *MELK*, *TIMELESS*, and *SAPCD2*) were closely involved in the molecular pathogenesis of LUAD. Furthermore, *GINS4* was directly regulated by these two miRNAs and the overexpression of *GINS4* facilitated LUAD cell aggressiveness. Based on the tumor-suppressive miRNA analysis, it was possible to identify miRNA target genes closely involved in the molecular pathogenesis of LUAD.

## Figures and Tables

**Table 1:** Downregulated miRNAs in LUAD clinical tissues by RNA sequencing.

MicroRNA	miRBase Accession No.	Guide or Passenger Strand	Log_2_ FC	*p*-Value	FDR
*hsa-miR-517b-3p*	MIMAT0002857	Guide strand	−4.00	<0.001	0.002
*hsa-miR-518a-3p*	MIMAT0002863	Guide strand	−3.46	<0.001	<0.001
*hsa-miR-551b-5p*	MIMAT0004794	Passenger strand	−3.39	0.004	0.022
*hsa-miR-523-5p*	MIMAT0005449	Passenger strand	−3.18	0.014	0.071
*hsa-miR-4703-3p*	MIMAT0019802	Guide strand	−3.15	<0.001	<0.001
*hsa-miR-6722-5p*	MIMAT0025853	Passenger strand	−3.12	<0.001	0.002
*hsa-miR-34c-5p*	MIMAT0000686	Guide strand	−3.11	0.030	0.129
*hsa-miR-486-5p*	MIMAT0002177	Guide strand	−3.11	0.002	0.009
*hsa-miR-218-1-3p*	MIMAT0004565	Passenger strand	−3.07	0.012	0.061
*hsa-miR-518e-5p*	MIMAT0005450	Passenger strand	−3.01	0.001	0.008
*hsa-miR-34c-3p*	MIMAT0004677	Passenger strand	−2.96	0.010	0.050
*hsa-miR-1208*	MIMAT0005873	Guide strand	−2.95	<0.001	0.002
*hsa-miR-4795-3p*	MIMAT0019969	Passenger strand	−2.95	<0.001	<0.001
*hsa-miR-4455*	MIMAT0018977	Guide strand	−2.92	<0.001	0.002
*hsa-miR-34b-3p*	MIMAT0004676	Guide strand	−2.90	0.027	0.119
*hsa-miR-603*	MIMAT0003271	Guide strand	−2.90	<0.001	<0.001
*hsa-miR-519a-3p*	MIMAT0002869	Guide strand	−2.90	0.002	0.012
*hsa-miR-486-3p*	MIMAT0004762	Passenger strand	−2.86	0.003	0.016
*hsa-miR-34b-5p*	MIMAT0000685	Passenger strand	−2.73	0.046	0.175
*hsa-miR-4532*	MIMAT0019071	Guide strand	−2.70	0.024	0.106
*hsa-miR-4655-3p*	MIMAT0019722	Passenger strand	−2.70	0.039	0.157
*hsa-miR-4281*	MIMAT0016907	Guide strand	−2.66	0.006	0.035
*hsa-miR-518f-3p*	MIMAT0002842	Guide strand	−2.66	0.014	0.068
*hsa-miR-6813-3p*	MIMAT0027527	Passenger strand	−2.65	0.001	0.007
*hsa-miR-940*	MIMAT0004983	Guide strand	−2.63	0.030	0.127
*hsa-miR-371b-3p*	MIMAT0019893	Passenger strand	−2.50	0.047	0.178
*hsa-miR-516b-5p*	MIMAT0002859	Guide strand	−2.50	0.022	0.100
*hsa-miR-4483*	MIMAT0019017	Guide strand	−2.36	0.034	0.141
*hsa-miR-523-3p*	MIMAT0002840	Guide strand	−2.34	0.023	0.106
*hsa-miR-758-5p*	MIMAT0022929	Passenger strand	−2.31	0.038	0.153
*hsa-miR-1258*	MIMAT0005909	Guide strand	−2.31	0.040	0.158
*hsa-miR-4529-5p*	MIMAT0019236	Passenger strand	−2.22	0.028	0.120
*hsa-miR-518c-3p*	MIMAT0002848	Guide strand	−2.16	0.027	0.117
*hsa-miR-6768-5p*	MIMAT0027436	Guide strand	−2.13	0.016	0.076
*hsa-miR-3622a-5p*	MIMAT0018003	Guide strand	−2.12	0.034	0.141
*hsa-miR-144-5p*	MIMAT0004600	Passenger strand	−2.12	0.018	0.086
*hsa-miR-373-3p*	MIMAT0000726	Guide strand	−2.09	0.038	0.152
*hsa-miR-451a*	MIMAT0001631	Guide strand	−2.07	0.038	0.153
*hsa-miR-144-3p*	MIMAT0000436	Guide strand	−2.06	0.024	0.107
*hsa-miR-4723-5p*	MIMAT0019838	Guide strand	−2.05	0.041	0.161
*hsa-miR-5011-5p*	MIMAT0021045	Passenger strand	−2.02	0.025	0.110

FDR, false discovery rate.

**Table 2:** Putative target genes regulated by *miR-486-5p* or *miR-486-3p* in A549 cells.

Gene ID	Gene Symbol	Gene Name	*miR-486-5p* Total Sites	*miR-486-3p* Total Sites	*miR-486-5p* Transfectant Log_2_ FC	*miR-486-3p* Transfectant Log_2_ FC
5141	*PDE4A*	Phosphodiesterase 4A	1	3	−2.25	−1.06
9783	*RIMS3*	Regulating synaptic membrane Exocytosis 3	1	1	−2.22	−0.67
79856	*SNX22*	Sorting nexin 22	2	1	−2.15	−2.67
4300	*MLLT3*	MLLT3 super-elongation complex subunit	1	2	−2.10	−1.18
2012	*EMP1*	Epithelial membrane protein 1	2	1	−2.06	−0.90
79628	*SH3TC2*	SH3 domain and tetratricopeptide repeats 2	3	1	−1.96	−1.97
195828	*ZNF367*	Zinc finger protein 367	1	1	−1.94	−1.10
115650	*TNFRSF13C*	TNF-receptor superfamily member 13C	1	5	−1.85	−0.61
84959	*UBASH3B*	Ubiquitin-associated and SH3 domain containing B	1	2	−1.62	−2.16
246243	*RNASEH1*	Ribonuclease H1	1	1	−1.60	−1.21
339768	*ESPNL*	Espin-like	1	2	−1.55	−1.46
220988	*HNRNPA3*	Heterogeneous nuclear ribonucleoprotein A3	1	2	−1.51	−1.20
9411	*ARHGAP29*	Rho GTPase-activating protein 29	2	1	−1.48	−3.37
81491	*GPR63*	G-protein-coupled receptor 63	2	2	−1.47	−0.82
56906	*THAP10*	THAP domain containing 10	1	1	−1.47	−1.63
54751	*FBLIM1*	Filamin-binding LIM protein 1	1	1	−1.46	−0.76
248	*ALPI*	Alkaline phosphatase, intestinal	1	3	−1.40	−1.09
122953	*JDP2*	Jun dimerization protein 2	2	3	−1.38	−1.73
6689	*SPIB*	Spi-B transcription factor	1	4	−1.37	−0.77
6722	*SRF*	Serum response factor	1	4	−1.32	−0.72
64710	*NUCKS1*	Nuclear casein kinase and cyclin-dependent kinase substrate 1	1	1	−1.30	−1.45
29920	*PYCR2*	Pyrroline-5-carboxylate reductase 2	1	1	−1.28	−1.40
81621	*KAZALD1*	Kazal-type serine peptidase-inhibitor domain 1	1	1	−1.27	−1.76
7433	*VIPR1*	Vasoactive intestinal peptide receptor 1	1	2	−1.26	−2.74
2304	*FOXE1*	Forkhead box E1	1	1	−1.21	−0.68
4288	*MKI67*	Marker of proliferation Ki-67	2	1	−1.21	−3.01
84296	*GINS4*	GINS complex subunit 4	1	1	−1.21	−2.72
6241	*RRM2*	Ribonucleotide reductase regulatory subunit M2	1	1	−1.21	−3.92
201292	*TRIM65*	Tripartite motif containing 65	1	1	−1.17	−0.65
57153	*SLC44A2*	Solute carrier family 44 member 2	1	4	−1.17	−1.67
2649	*NR6A1*	Nuclear receptor subfamily 6 group A member 1	1	4	−1.11	−0.96
6720	*SREBF1*	Sterol regulatory element-binding transcription factor 1	1	1	−1.09	−1.68
339834	*CCDC36*	Coiled-coil domain containing 36	2	1	−1.08	−1.65
57506	*MAVS*	Mitochondrial antiviral signaling protein	4	4	−1.07	−0.51
85014	*TMEM141*	Transmembrane protein 141	1	2	−1.06	−1.54
283349	*RASSF3*	Ras association domain family member 3	1	1	−1.04	−1.81
160518	*DENND5B*	DENN domain containing 5B	1	1	−1.04	−0.65
92126	*DSEL*	Dermatan sulfate epimerase-like	1	1	−1.03	−0.72
3070	*HELLS*	Helicase, lymphoid specific	1	2	−1.02	−1.90
11051	*NUDT21*	Nudix hydrolase 21	1	2	−1.01	−1.06
345557	*PLCXD3*	Phosphatidylinositol-specific phospholipase C X domain containing 3	1	1	−1.00	−2.20
11113	*CIT*	Citron rho-interacting serine/threonine kinase	1	1	−1.00	−2.89
145508	*CEP128*	Centrosomal protein 128	1	1	−0.99	−2.54
3707	*ITPKB*	Inositol-trisphosphate 3-kinase B	1	2	−0.97	−1.07
59269	*HIVEP3*	HIVEP zinc finger 3	1	3	−0.97	−1.29
81563	*C1orf21*	Chromosome 1 open reading frame 21	1	1	−0.94	−0.66
84515	*MCM8*	Minichromosome maintenance 8 homologous recombination repair factor	1	1	−0.94	−1.80
93129	*ORAI3*	ORAI calcium release-activated calcium modulator 3	1	1	−0.94	−0.62
119	*ADD2*	Adducin 2	2	1	−0.92	−4.18
5939	*RBMS2*	RNA binding motif single-stranded interacting protein 2	1	2	−0.90	−0.51
55512	*SMPD3*	Sphingomyelin phosphodiesterase 3	1	3	−0.89	−2.43
9833	*MELK*	Maternal embryonic leucine zipper kinase	1	1	−0.86	−2.20
30815	*ST6GALNAC6*	ST6 N-acetylgalactosaminide alpha-2,6-sialyltransferase 6	1	2	−0.85	−0.73
64077	*LHPP*	Phospholysine phosphohistidine inorganic pyrophosphate phosphatase	2	3	−0.84	−1.38
6526	*SLC5A3*	Solute carrier family 5 member 3	2	1	−0.83	−0.78
10272	*FSTL3*	Follistatin-like 3	1	2	−0.80	−0.71
10613	*ERLIN1*	ER lipid raft-associated 1	1	2	−0.80	−1.48
651746	*ANKRD33B*	Ankyrin repeat domain 33B	1	3	−0.79	−1.28
26468	*LHX6*	LIM homeobox 6	1	1	−0.78	−1.80
196743	*PAOX*	Polyamine oxidase	1	3	−0.77	−1.82
8624	*PSMG1*	Proteasome assembly chaperone 1	2	2	−0.76	−1.27
57546	*PDP2*	Pyruvate dehyrogenase phosphatase catalytic subunit 2	1	3	−0.75	−1.31
10592	*SMC2*	Structural maintenance of chromosomes 2	2	1	−0.74	−1.92
54475	*NLE1*	Notchless homolog 1	1	2	−0.73	−1.95
8573	*CASK*	Calcium/calmodulin-dependent serine protein kinase	2	1	−0.72	−0.51
153443	*SRFBP1*	Serum response factor-binding protein 1	1	1	−0.72	−0.65
10217	*CTDSPL*	CTD small-phosphatase-like	1	1	−0.72	−0.76
81029	*WNT5B*	Wnt family member 5B	1	1	−0.70	−2.63
60312	*AFAP1*	Actin filament-associated protein 1	1	3	−0.70	−1.66
23216	*TBC1D1*	TBC1 domain family member 1	1	1	−0.68	−1.20
7301	*TYRO3*	TYRO3 protein tyrosine kinase	1	1	−0.67	−1.52
2000	*ELF4*	E74-like ETS transcription factor 4	1	2	−0.67	−1.49
5064	*PALM*	Paralemmin	1	3	−0.67	−1.87
79622	*SNRNP25*	Small nuclear ribonucleoprotein U11/U12 subunit 25	1	1	−0.66	−0.71
64399	*HHIP*	Hedgehog interacting protein	1	2	−0.65	−0.65
23075	*SWAP70*	Switching B-cell complex subunit SWAP70	1	1	−0.64	−0.88
118980	*SFXN2*	Aideroflexin 2	1	1	−0.64	−2.07
4087	*SMAD2*	SMAD family member 2	1	1	−0.64	−0.86
317762	*CCDC85C*	Coiled-coil domain containing 85C	1	1	−0.63	−2.02
84440	*RAB11FIP4*	RAB11 family interacting protein 4	1	8	−0.60	−0.73
131566	*DCBLD2*	Discoidin, CUB and LCCL domain containing 2	1	1	−0.60	−2.78
4771	*NF2*	Neurofibromin 2	1	1	−0.59	−1.88
84083	*ZRANB3*	Zinc finger RANBP2-type containing 3	2	1	−0.59	−1.62
8914	*TIMELESS*	Timeless circadian regulator	1	1	−0.58	−1.65
8125	*ANP32A*	Acidic nuclear phosphoprotein 32 family member A	1	2	−0.57	−1.57
25937	*WWTR1*	WW domain containing transcription regulator 1	2	2	−0.57	−1.46
255104	*TMCO4*	Transmembrane and coiled-coil domains 4	1	1	−0.56	−1.09
51308	*REEP2*	Receptor accessory protein 2	1	2	−0.56	−1.45
84908	*FAM136A*	Family with sequence similarity 136 member A	1	1	−0.56	−0.82
25961	*NUDT13*	Nudix hydrolase 13	1	1	−0.55	−1.49
81839	*VANGL1*	VANGL planar cell polarity protein 1	2	2	−0.55	−0.78
54820	*NDE1*	NudE neurodevelopment protein 1	1	1	−0.55	−1.40
117584	*RFFL*	Ring finger and FYVE-like domain containing E3 ubiquitin protein ligase	1	1	−0.54	−0.73
6839	*SUV39H1*	Suppressor of variegation 3–9 homolog 1	1	3	−0.53	−3.23
154810	*AMOTL1*	Angiomotin-like 1	1	2	−0.51	−1.09
79096	*C11orf49*	Chromosome 11 open reading frame 49	1	2	−0.51	−1.84
89958	*SAPCD2*	Suppressor APC domain containing 2	1	4	−0.51	−1.98
9801	*MRPL19*	Mitochondrial ribosomal protein L19	1	2	−0.51	−0.78
84948	*TIGD5*	Tigger transposable element-derived 5	1	1	−0.50	−0.91

**Table 3:** *GINS4*-mediated pathways by Gene Set Enrichment Analysis (GSEA).

Pathway	Enrichment Score	Normalized Enrichment Score	*p*-Value	FDR
KEGG_CELL_CYCLE	0.74	2.91	<0.001	<0.001
KEGG_DNA_REPLICATION	0.83	2.61	<0.001	<0.001
KEGG_HOMOLOGOUS_RECOMBINATION	0.76	2.24	<0.001	<0.001
KEGG_MISMATCH_REPAIR	0.77	2.22	<0.001	<0.001
KEGG_OOCYTE_MEIOSIS	0.58	2.20	<0.001	<0.001
KEGG_SPLICEOSOME	0.56	2.17	<0.001	<0.001
KEGG_PROTEASOME	0.61	2.02	<0.001	<0.001
KEGG_P53_SIGNALING_PATHWAY	0.53	1.92	<0.001	0.001
KEGG_NUCLEOTIDE_EXCISION_REPAIR	0.58	1.88	<0.001	0.002
KEGG_BASAL_TRANSCRIPTION_FACTORS	0.59	1.86	<0.001	0.002

FDR, false discovery rate.

## Data Availability

Publicly available datasets were analyzed in this study. These data can be accessed here: https://www.ncbi.nlm.nih.gov/geo/query/acc.cgi?acc=GSE230229 (accessed on 19 June 2023) and https://www.ncbi.nlm.nih.gov/geo/query/acc.cgi?acc=GSE230056 (accessed on 19 June 2023).

## Supplementary Materials

The following supporting information can be downloaded at: https://www.mdpi.com/article/10.3390/cells12141885/s1, Figure S1: suppression of mRNA expression levels of 7 target genes (*MKI67*, *GINS4*, *RRM2*, *HELLS*, *MELK*, *TIMELESS*, and *SAPCD2*) by ectopic expressions of *miR-486-5p* or *miR-486-3p* in A549 cells; Figure S2: full-sized images of Western blot analysis (*GINS4* antibody) following ectopic expressions of *miR-486-5p* or *miR-486-3p* in A549 and H1299 cells; Figure S3: suppression of mRNA expression levels of *GINS4* by the transfection of siRNAs (si*GINS4*-1, si*GINS4*-2, and si*GINS4*-3) in A549 and H1299 cells; Figure S4: full-sized images of Western blotting (*GINS4* antibody) following the transfection of siRNAs (si*GINS4*-1, si*GINS4*-2, and si*GINS4*-3) in A549 and H1299 cells; Table S1: clinical features of LUAD patients created by the miRNA expression signature; Table S2: reagents used in this study; Table S3: information of tissues by immunostaining; Table S4: human genome-matched sequence reads.
