# Supplementary material for: The Molecular Pathogenesis of Tumor-Suppressive miR-486-5p and miR-486-3p Target Genes: GINS4 Facilitates Aggressiveness in Lung Adenocarcinoma
Source: Cells. 2023 Jul 18;12(14):1885. doi: 10.3390/cells12141885 (PMC10378275; doi:10.3390/cells12141885)
Supplement: Supplementary file 1 [file cells-12-01885-s001.zip › Cells Molecular targets controlled by pre-miR-486_ in lung adenocarcinoma figure Supplement.pptx]

## Slide 1
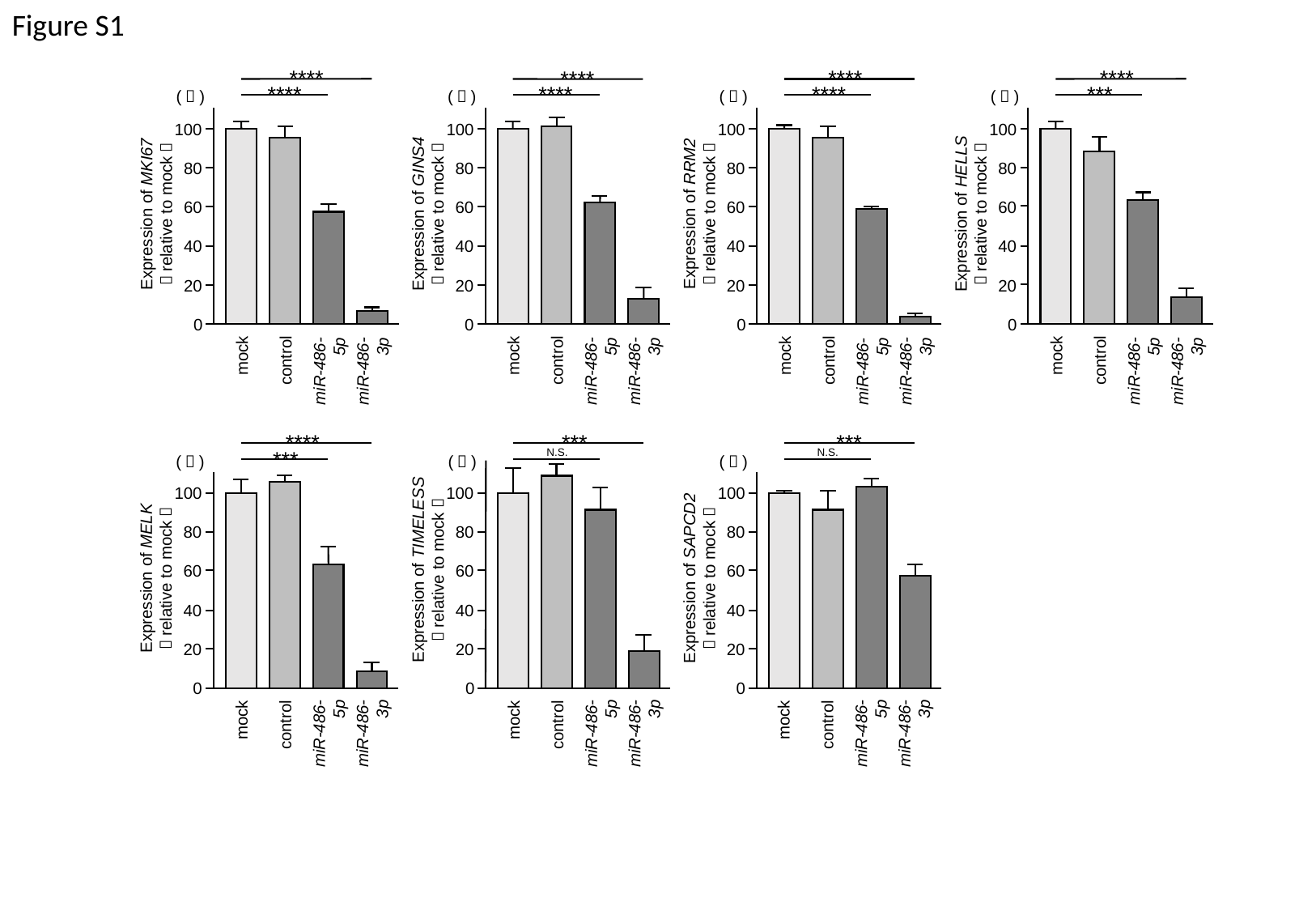

Figure S1
****
****
(％)
100
80
Expression of MKI67
（relative to mock）
60
40
20
0
mock
control
miR-486-3p
miR-486-5p
****
****
(％)
100
80
Expression of RRM2
（relative to mock）
60
40
20
0
mock
control
miR-486-3p
miR-486-5p
****
***
(％)
100
80
Expression of HELLS
（relative to mock）
60
40
20
0
mock
control
miR-486-3p
miR-486-5p
****
****
(％)
100
80
Expression of GINS4
（relative to mock）
60
40
20
0
mock
control
miR-486-3p
miR-486-5p
****
***
(％)
100
80
Expression of MELK
（relative to mock）
60
40
20
0
mock
control
miR-486-5p
miR-486-3p
***
N.S.
(％)
100
80
Expression of TIMELESS
（relative to mock）
60
40
20
0
mock
control
miR-486-5p
miR-486-3p
***
N.S.
(％)
100
80
Expression of SAPCD2
（relative to mock）
60
40
20
0
mock
control
miR-486-5p
miR-486-3p

## Slide 2
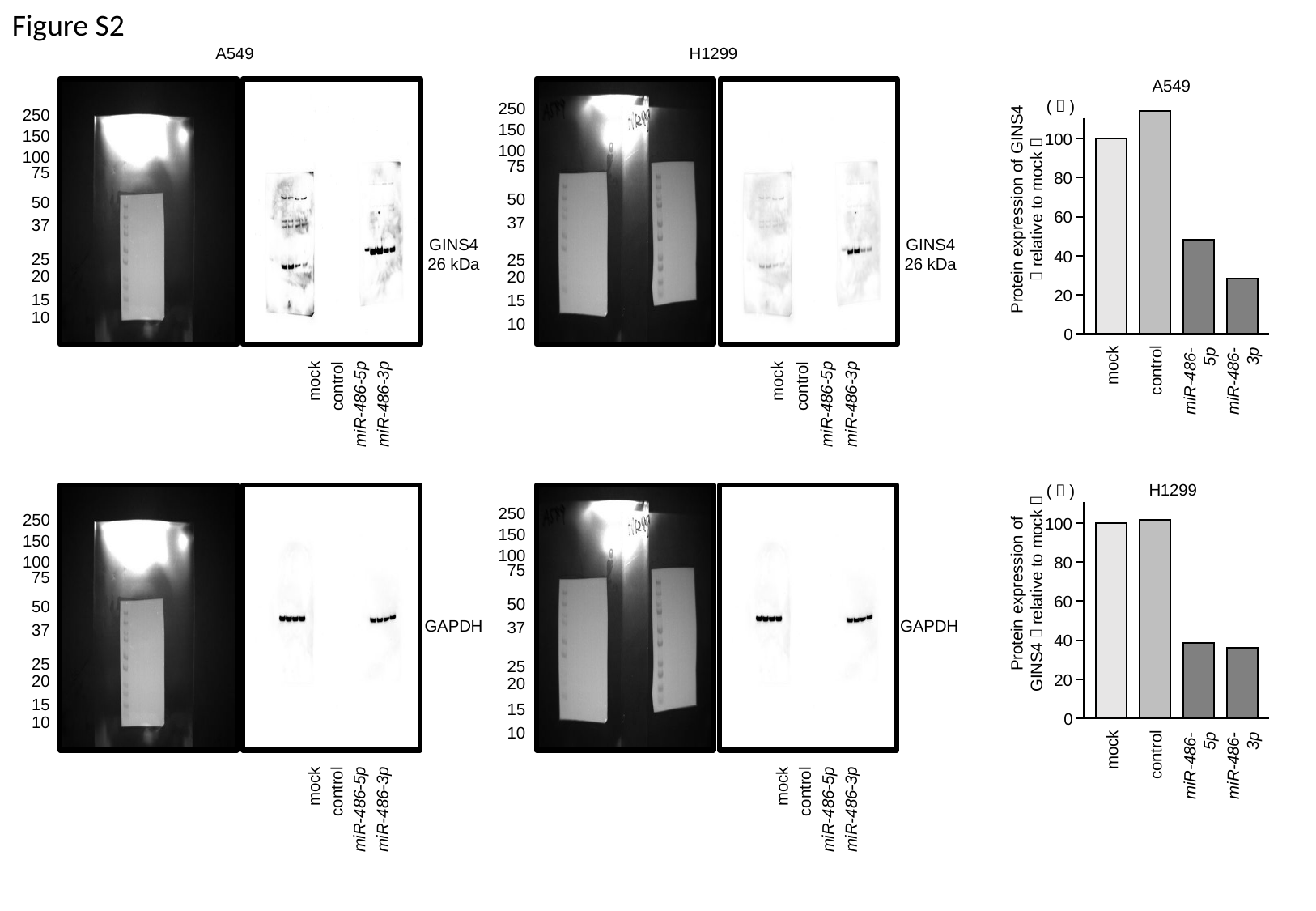

Figure S2
A549
H1299
A549
(％)
250
250
150
150
100
100
100
75
75
80
50
Protein expression of GINS4
（relative to mock）
50
60
37
37
GINS4
26 kDa
GINS4
26 kDa
40
25
25
20
20
20
15
15
10
10
0
mock
control
miR-486-3p
miR-486-5p
mock
control
miR-486-5p
miR-486-3p
mock
control
miR-486-5p
miR-486-3p
H1299
(％)
250
250
100
150
150
100
100
80
75
75
Protein expression of GINS4（relative to mock）
60
50
50
GAPDH
GAPDH
37
37
40
25
25
20
20
20
15
15
0
10
10
mock
control
miR-486-3p
miR-486-5p
mock
control
miR-486-5p
miR-486-3p
mock
control
miR-486-5p
miR-486-3p

## Slide 3
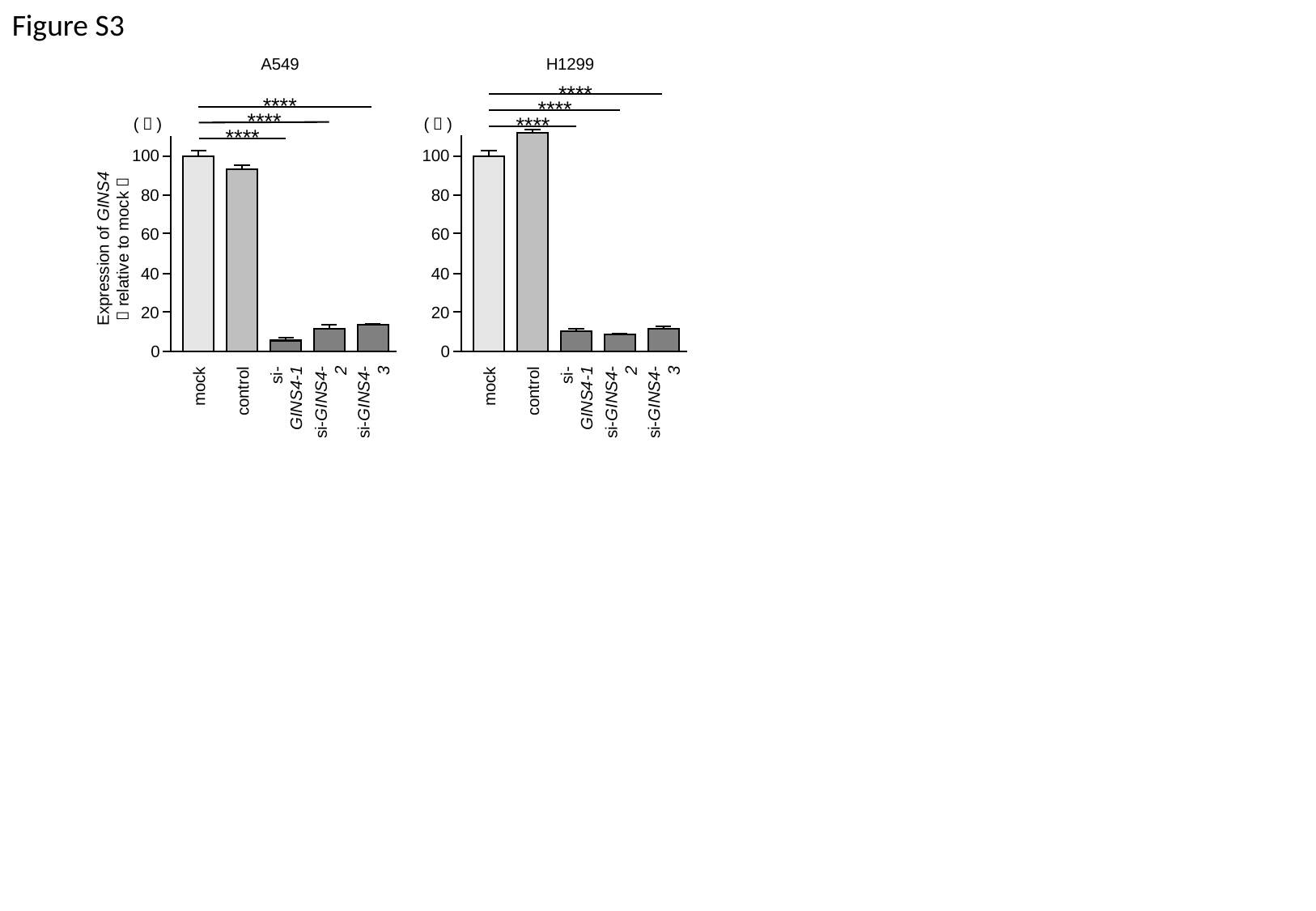

Figure S3
A549
****
****
(％)
****
100
80
60
Expression of GINS4
（relative to mock）
40
20
0
mock
control
si-GINS4-1
si-GINS4-2
si-GINS4-3
H1299
****
****
****
(％)
100
80
60
40
20
0
mock
control
si-GINS4-1
si-GINS4-2
si-GINS4-3

## Slide 4
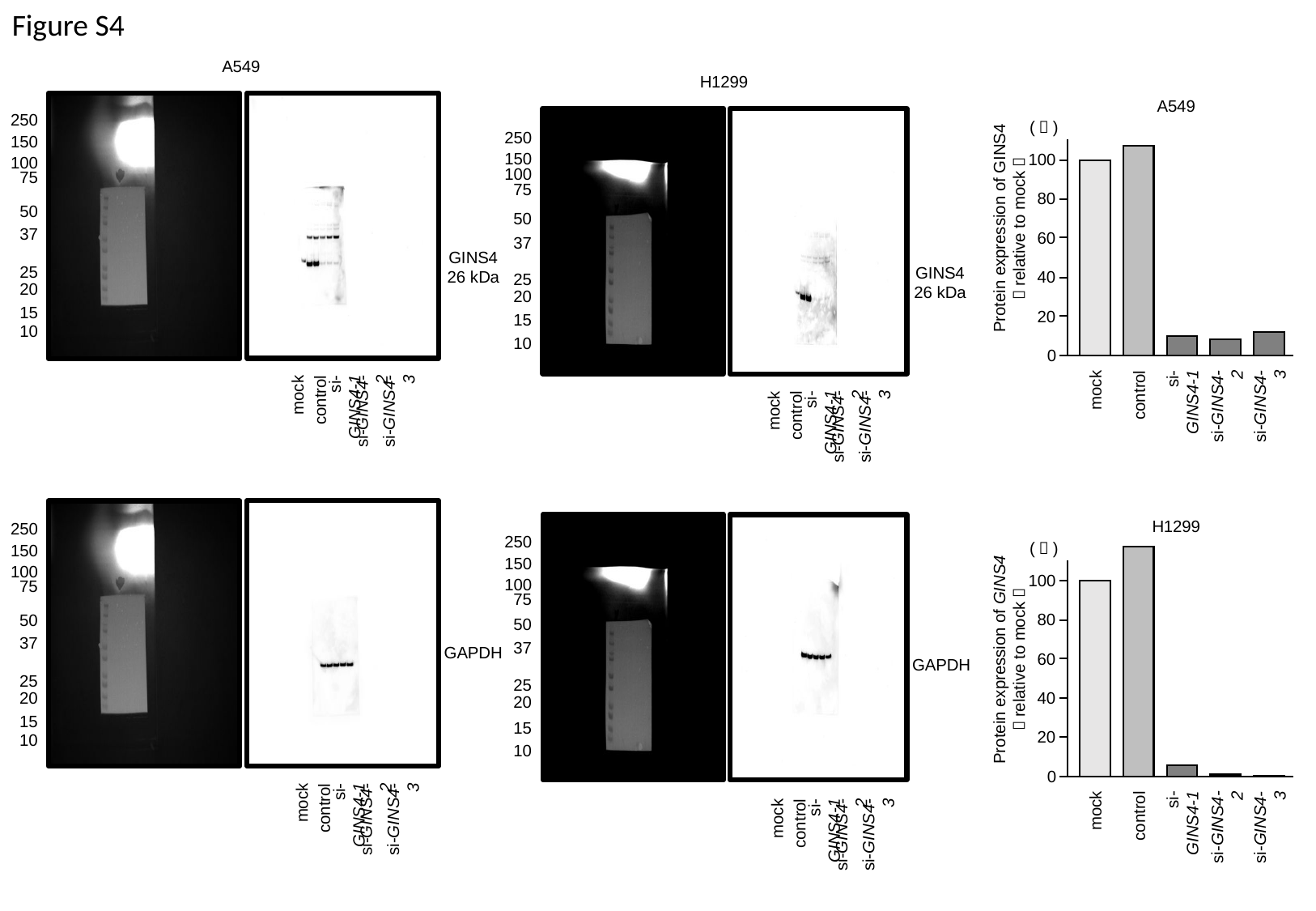

Figure S4
A549
H1299
A549
250
(％)
250
150
150
100
100
100
75
75
80
50
50
Protein expression of GINS4
（relative to mock）
37
60
37
GINS4
26 kDa
25
GINS4
26 kDa
40
25
20
20
15
20
15
10
10
0
mock
control
si-GINS4-1
si-GINS4-2
si-GINS4-3
mock
control
si-GINS4-1
si-GINS4-2
si-GINS4-3
mock
control
si-GINS4-1
si-GINS4-2
si-GINS4-3
H1299
250
250
(％)
150
150
100
100
100
75
75
80
50
50
37
37
Protein expression of GINS4
（relative to mock）
GAPDH
60
GAPDH
25
25
20
40
20
15
15
20
10
10
0
mock
control
si-GINS4-1
si-GINS4-2
si-GINS4-3
mock
control
si-GINS4-1
si-GINS4-2
si-GINS4-3
mock
control
si-GINS4-1
si-GINS4-2
si-GINS4-3
